# Cancer-Associated Intermediate Conductance Ca^2+^-Activated K^+^ Channel K_Ca_3.1

**DOI:** 10.3390/cancers11010109

**Published:** 2019-01-17

**Authors:** Corinna J. Mohr, Friederike A. Steudel, Dominic Gross, Peter Ruth, Wing-Yee Lo, Reiner Hoppe, Werner Schroth, Hiltrud Brauch, Stephan M. Huber, Robert Lukowski

**Affiliations:** 1 Department of Pharmacology, Toxicology and Clinical Pharmacy, Institute of Pharmacy, University of Tuebingen, 72076 Tuebingen, Germany; corinna.mohr@uni-tuebingen.de (C.J.M.); friederike.steudel@googlemail.com (F.A.S.); dominic.gross@uni-tuebingen.de (D.G.); peter.ruth@uni-tuebingen.de (P.R.); 2 Dr. Margarete Fischer-Bosch-Institute of Clinical Pharmacology, 70376 Stuttgart, Germany; Wing-Yee.Lo@ikp-stuttgart.de (W.-Y.L.); Reiner.Hoppe@ikp-stuttgart.de (R.H.); Werner.Schroth@ikp-stuttgart.de (W.S.); Hiltrud.Brauch@ikp-stuttgart.de (H.B.); 3 University of Tuebingen, 72076 Tuebingen, Germany; 4 German Cancer Consortium (DKTK), German Cancer Research Center (DKFZ), 69120 Heidelberg, Germany; 5 Department of Radiation Oncology, University of Tuebingen, 72076 Tuebingen, Germany; stephan.huber@uni-tuebingen.de

**Keywords:** K_Ca_3.1, intermediate conductance calcium-activated K^+^ channel, BK, big conductance Ca^2+^- and voltage-activated K^+^ channels, TRAM-34, (1-[(2-chlorophenyl) diphenylmethyl]-pyrazole, 1-EBIO, 1-Ethyl-1,3-dihydro-2H-benzimidazol-2-one, E2, 17β-estradiol

## Abstract

Several tumor entities have been reported to overexpress K_Ca_3.1 potassium channels due to epigenetic, transcriptional, or post-translational modifications. By modulating membrane potential, cell volume, or Ca^2+^ signaling, K_Ca_3.1 has been proposed to exert pivotal oncogenic functions in tumorigenesis, malignant progression, metastasis, and therapy resistance. Moreover, K_Ca_3.1 is expressed by tumor-promoting stroma cells such as fibroblasts and the tumor vasculature suggesting a role of K_Ca_3.1 in the adaptation of the tumor microenvironment. Combined, this features K_Ca_3.1 as a candidate target for innovative anti-cancer therapy. However, immune cells also express K_Ca_3.1 thereby contributing to T cell activation. Thus, any strategy targeting K_Ca_3.1 in anti-cancer therapy may also modulate anti-tumor immune activity and/or immunosuppression. The present review article highlights the potential of K_Ca_3.1 as an anti-tumor target providing an overview of the current knowledge on its function in tumor pathogenesis with emphasis on vasculo- and angiogenesis as well as anti-cancer immune responses.

## 1. Introduction

The K_Ca_3.1 channel, also known as SK4 or IK, is activated by a rise of the intracellular Ca^2+^ concentration [Ca^2+^]_i_. Co-assembly of four K_Ca_3.1 pore-forming α subunits is required to form a functional channel. The basis for their Ca^2+^ sensitivity is conferred by a constitutively bound calmodulin in the C-terminal tail of each α subunit. Through binding to calmodulin, Ca^2+^ induces a conformational change that permits channel opening. The single channel conductance of the K_Ca_3.1 ranges between 20 to 80 pS, hence intermediary between the single channel conductance of related Ca^2+^-activated K^+^ channels with either small (SK1-3 channels with 5–20 pS) or big conductance (100–300 pS) [1,2,3]. K_Ca_3.1 channels conduct K^+^ across the membrane of excitable and non-excitable cells, but they lack the typical features of a voltage-sensing domain. Additionally, K_Ca_3.1 activity is also regulated by histidine phosphorylation [4,5,6]. 

Under physiological conditions, K_Ca_3.1 channels are expressed in epithelial, endothelial, and hematopoietic cells [7], whereas their presence in excitable cells such as central neurons and cardiomyocytes has only recently been recognized [8,9,10]. In secretory epithelia such as lung and intestine, K_Ca_3.1 channels contribute to the normal electrochemical gradient for transepithelial secretion of Cl^−^, Na^+^ and H_2_O [11]. K_Ca_3.1 currents were detected in vascular smooth muscle cells derived from murine arteries subjected to the wire injury model of restenosis, but not in those of uninjured control vessels. Importantly, neointima formation was significantly impaired after targeted disruption of K_Ca_3.1 [12]. Basic fibroblast growth factor- (bFGF) and vascular endothelial growth factor (VEGF)-treated human endothelial cells also upregulated K_Ca_3.1 implying a role in the formation of new blood vessels [13]. Finally, a lack of K_Ca_3.1 causes mild hypertension in the dark phase suggesting a significant role in blood pressure control during physical activity [14]. With regard to its role in the immune system, upregulation and activation of K_Ca_3.1 in T cells in response to antigens and mitogens are well established. In this context, K_Ca_3.1 possibly acts upon a nucleoside diphosphate kinase B-mediated histidine phosphorylation to promote T cell activation ultimately resulting in their clonal expansion [15]. A number of additional K_Ca_3.1 functions point to a role in the migration of lipopolysaccharide-(LPS) activated dendritic cells (DCs) [16], proper mast cell activation after IgE binding [17], the pathogenesis of airway inflammation and remodelling in allergic asthma [18], the prevention of hyper-responsiveness to acute stress by modulating the release of corticotropin from the anterior pituitary gland [19], the processing of pain induced by noxious chemical stimuli [20], neuroinflammation in murine stroke models [21], and renal fibroblast proliferation induced by unilateral urethral obstruction in mice [22]. Finally, a number of studies have revealed that K_Ca_3.1 is involved in Ca^2+^-dependent K^+^ efflux from erythrocytes, which in combination with Cl^−^ and H_2_O movement mediates cell shrinkage, a phenomenon referred to as ‘Gardos effect’ [23]. 

It is becoming increasingly clear that K_Ca_3.1-dependent signaling pathways affect the immune system and mechanisms of cell proliferation and migration; hence, it is not surprising that K_Ca_3.1 plays a role in cancer development and progression. While the details of tumor-related K_Ca_3.1 functions are subject to continued investigation, K_Ca_3.1 channels have emerged as promising targets for immunomodulation in drug-resistant cancers. This review extends previous important works by others [24,25,26] in highlighting the multitude of K_Ca_3.1 physiological functions and their complex role in cancer. 

## 2. Tumor Cell-Specific Functions of K_Ca_3.1 

### 2.1. Molecular Markers and Regulation of KCNN4

Among the known influences that regulate the expression of the K_Ca_3.1-encoding *KCNN4* gene are constitutional, epigenetic, and post-transcriptional variations. In this paragraph, we describe some of these effects on *KCNN4* expression as they have been reported for a number of different cancer types including breast, lung, endometrial, and pancreatic cancer.

**Figure 1:**
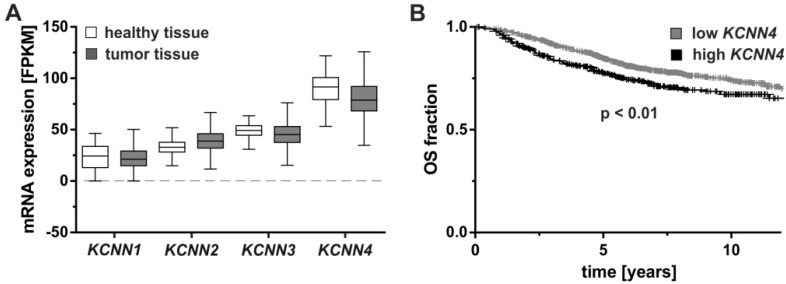
*KCNN4* mRNA expression levels in breast cancer and their association with patient survival. (**A**) mRNA expression levels of *KCNN1-4* coding for SK1-SK3 and K_Ca_3.1 were compared between healthy and breast tumor tissues, measured by RNA sequencing as fragments per kilobase of transcript per million mapped reads (FPKM). Data obtained from The Cancer Genome Atlas [30] revealed no significant difference in a Kruskal–Wallis test with Dunn’s test for multiple comparisons (α = 0.05 for *n* = 113 healthy and *n* = 1095 breast tumor tissues). (**B**) In the Kaplan–Meier plotter [31], significantly prolonged overall survival (OS) was associated with low *KCNN4* mRNA levels. Groups were statistically compared by log-rank test (hazard ratio = 1.37 (confidence interval 1.08–1.72) for *n* = 1030 low and *n* = 372 high *KCNN4*-expressing tumors).

Sequence variations known as single nucleotide polymorphisms (SNP) may impact on gene expression when located in regulatory sites such as non-coding regions. It is therefore of interest that the SNP rs3760982 located at the intergenic region of *KCNN4* and *LYPD5* (LY6/PLAUR Domain Containing 5, metastasis-associated protein) on chromosome 19q13.31 has been shown to be associated with breast cancer risk [27], a finding that was corroborated in large scale genome wide association studies (GWAS) using data sets of more than 200,000 patients and controls (P = 1.4 × 10^−16^ [28]). Notably, the association is strongest in patients with tumors expressing estrogen receptors (ER; P = 4 × 10^−14^) who are predestined to receive anti-hormonal treatment. A number of *KCNN4* SNPs reside within the first intron of the gene, some of which may be associated as well with ER-positive breast cancer risk [29], however, whether or not dysregulated *KCNN4* expression is the cause of this risk association and which role the genetic control of the K_Ca_3.1 channel plays for breast cancer development is not clear. At the tumor level, the degree of *KCNN4* mRNA expression is potentially useful to stratify breast cancer patients into those with shorter and longer survival time. Data from The Cancer Genome Atlas suggests no difference in *KCNN4* mRNA expression between normal and breast tumor tissue [30] (Figure 1A), however, higher *KCNN4* expression in the tumor tissue might modify patient outcome as indicated by the shorter overall survival in Kaplan–Meier analysis [31] (Figure 1B). In addition, high *KCNN4*-mRNA levels were also associated with a lower overall survival, shorter progression-free survival, and a high metastatic potential of patients with clear cell renal cell carcinoma (ccRCC) suggesting that K_Ca_3.1 may be of prognostic value in ccRCC [32].

Epigenetically, the regulation of gene expression is influenced by chromatin modifications such as acetylation or methylation as well as DNA methylation in the proximity of gene regions. In non-small cell lung cancer, *KCNN4* promoter hypomethylation has been observed particularly in advanced-stage tumors. *KCNN4* promoter hypomethylation was accompanied by an increase in mRNA expression when compared to normal lung tissue, which was also associated with shorter progression-free and overall survival. Notably, this observation in patients is supported by findings in a model of A549 lung adenocarcinoma cells in which higher *KCNN4* mRNA and K_Ca_3.1 protein expression levels, as well as aggressive tumor cell behavior, were observed. Functional tests revealed decreased proliferation and migration upon K_Ca_3.1 inhibition with TRAM-34. Moreover, A549 xenografts in nude mice showed attenuated tumor growth when treated with the K_Ca_3.1 inhibitor senicapoc [33].

The influence of post-transcriptional control via microRNAs (miRNAs) on the expression of K_Ca_3.1 is not well understood. miRNAs are a large family of highly conserved, small non-protein-coding RNA molecules that function as critical regulators of gene expression by triggering either translational repression or degradation of their target mRNAs [34]. Individual miRNAs act either as tumor suppressors by repressing oncogene expression or as oncogenes by repressing tumor suppressor genes. Although K_Ca_3.1 has been observed to be upregulated in pancreatic, breast, and endometrial cancers which affects tumor progression [35,36,37], not much is known about the underlying dysregulation of miRNAs. Yet, in angiosarcoma, miR-497-5p acts in a tumor-suppressive mode as it inhibited cell proliferation and invasion via downregulation of K_Ca_3.1, an observation that highlights both, the regulatory miRNA and the targeted K_Ca_3.1 channel as potential new treatment targets [38]. Similarly, miR-16-5p and miR-375 were identified to have the potential to modulate K_Ca_3.1 expression [39]. MiR-16-5p was among the first downregulated miRNAs identified in chronic lymphocytic leukemia due to frequent deletions [40] and moreover gained wider attention as a regulator of anti-apoptotic BCL2 in prostate and breast cancer [41,42] as well as breast cancer development [43]. Notably, miRNA-16-5p was repressed in MCF-7 breast cancer cells upon 17β-estradiol (E2) treatment, an effect that could be rescued using mimics in order to inhibit E_2_-induced cell proliferation [44]. The role of K_Ca_3.1 in this process is currently not known. In summary, the available information on K_Ca_3.1 regulation in cancer deserves further attention. 

### 2.2. Tumorigenesis

Cell division is a highly conserved and tightly controlled process that ensures the replication of DNA and its segregation into daughter cells. In tumor cells, control elements of the cell cycle such as growth factors and their receptors or growth-promoting cyclins are often dysregulated due to genetic aberrations. In combination with genetic aberrations affecting tumor suppressor genes, a number of alterations within a single cell may drive uncontrolled proliferation [45,46,47]. Important but often neglected features of the cell cycle are changes in transmembrane ion flux causing fluctuations of the electrical membrane potential or regulatory changes in cell volume. By modulating these processes, K_Ca_3.1 channels may contribute to the abnormal proliferation of tumor cells [47,48,49]. 

Changes in the cellular resting membrane potential (V_m_) are essential for cell cycle progression. When compared to excitable cells, V_m_ changes in tumor cells occurring during the cell cycle are usually slower and smaller [48,50]. In contrast to proliferating cells, a constant resting V_m_ is seen for example in striated muscle cells or neurons, which are usually associated with little or no mitotic activity. Activation of K^+^ channels in the plasma membrane has been shown to cause the more negative membrane potential necessary to initiate this G_1_/S transition [51]. Hyperpolarization of V_m_ is also required for G_1_/S transition, DNA synthesis, and progression through S phase, while V_m_ depolarization precedes G_2_/M progression and mitosis entry [47,49]. Accordingly, altered expression and activity of certain K^+^ channels throughout the different cell cycle phases have been observed [36,48,49,52,53,54]. Studies utilizing a number of K^+^ channel modulators further emphasize that both temporal and spatial changes in K^+^ channel activity play a crucial role for the transition from G_0_ into G_1_. For example, cell cycle progression was prevented upon blockade of the ATP-sensitive K^+^ channel or the human Ether-A-Go-Go-Related Protein 1 (*KCNH2*) in MCF-7 breast tumor cells and in different leukemia cell lines [36,51,52,55,56,57]. With regard to K_Ca_3.1 blockade by the antifungal imidazole, clotrimazole induced MCF-7 cell depolarization and prevented G_1_/S transition [36]. Furthermore, clotrimazole as well as its more specific analogue TRAM-34 arrested HEC-1-A endometrial cancer cells in G_0_/G_1_ phase and suppressed tumor development in nude mice [37]. Moreover, TRAM-34 as well as an RNAi-mediated depletion of K_Ca_3.1 increased the expression of the cyclin-dependent kinase inhibitor p21 important for blockade of G_1_/S transition and thus suppressed the proliferation of different prostate cancer cell lines. In contrast, the K_Ca_3.1 opener 1-EBIO evoked a clotrimazole-sensitive and concentration-dependent increase in the mitotic cell division [58,59]. In HepG2 hepatocellular carcinoma cells, anti-tumor growth effects of TRAM-34 were linked to a downregulation of ERα and nuclear factor-κB [60,61]. Interestingly, an overexpression of K_Ca_3.1 in the human MDA-MB-231 breast cancer cell line promoted the oncogenic cell growth in an in vivo xenograft model but not in vitro [62]. As the V_m_ of MDA-MB-231 cells was not altered by K_Ca_3.1 overexpression, pro-tumor functions of this channel in vivo seem to require a crosstalk with microenvironmental factors and/or other signals from non-tumor cells.

The Ca^2+^ dependence of K_Ca_3.1 activation directly links this channel to an important second messenger and various Ca^2+^ effector proteins regulating proliferation [63,64]. Ca^2+^ oscillations occur during G_1_ phase, G_1_/S and G_2_/M transitions as well as between metaphase and anaphase [64,65,66,67]. Cell cycle progression depends on regulated Ca^2+^ entry. Alterations in [Ca^2+^]_i_ have, therefore, been associated with abnormal activation of mitogenic pathways in various cancer cell types [65,68]. Through their effect on V_m_, K^+^ channels such as K_Ca_3.1 increase the driving force for Ca^2+^ influx and thus generate robust [Ca^2+^]_i_ signals to finally promote tumor cell proliferation [69]. Accordingly, in prostate and pancreatic cancer cells, the Ca^2+^ influx through transient receptor potential (TRP) vanilloid subfamily member 6 (TRPV6) channels is abolished by blockade of hyperpolarizing K_Ca_3.1 currents. In contrast, escalating extracellular Ca^2+^ used to artificially increase the driving force for Ca^2+^ entry stimulated cell proliferation even in the presence of K_Ca_3.1 channel blockers [35,59]. In MCF-7 breast cancer cells, K_Ca_3.1 and Ca^2+^-permeable canonical TRP subtype 1 (TRPC1) channels accumulate during G_1_ phase allowing them to interact and regulate basal Ca^2+^ entry [36,70]. Serum-containing growth factors evoked Ca^2+^ signals and transition to S phase was suppressed by pharmacological or genetic K_Ca_3.1 blockade in murine breast cancer cells [71]. Recent evidence suggests that ionizing radiation (IR) activates IK channels in glioblastoma cells due to an increase in [Ca^2+^]_i_. Accordingly, K_Ca_3.1 inhibition by TRAM-34 suppressed the clonogenic survival of irradiated but not that of unirradiated T98G and U87MG glioblastoma cells. In vivo, co-treatment with TRAM-34 increased the response of an ectopic glioblastoma mouse model to fractionated cancer radiotherapy [72]. 

Finally, K^+^ channel pathways may be directly linked to mitogens such as cyclins and immediate early genes. At least in parts, these non-canonical interactions seem to occur independently from K^+^ permeability changes via yet unknown mechanisms [47,48,49,68,71,73,74,75,76]. In K_Ca_3.1 gene-targeted murine breast cancer cells stimulated with serum, we recently confirmed a significant suppression of c-fos and c-jun mRNA levels. This finding is in accordance with the anti-proliferative and checkpoint functions attributed to K_Ca_3.1 activity [71]. However, K^+^ permeability and immediate early gene expression have not been assessed directly in K_Ca_3.1-positive versus -negative breast tumor cells and thus the impact of this channel on non-canonical pathways remains largely unclear.

Some studies also challenge the anti-tumorigenic potential of targeting K_Ca_3.1 and its critical role for the proliferation of cancer cells [77,78]. However, it is one serious limitation of the available reports that the applied pharmacological and/or siRNA approaches produce side effects owing to off-target actions. Accordingly, concerns were raised regarding the use of TRAM-34 because this ‘specific’ K_Ca_3.1 blocker was shown to stimulate the proliferation of breast cancer cells via the activation of ERs [79] and, in addition, Agarwal and colleagues suggested that TRAM-34 in low micromolar concentrations may also inhibit multiple cytochrome P450 isoforms [80]. We believe that these discrepancies regarding the proliferative and pharmacological properties of K_Ca_3.1 in cancer cells should be resolved by proof-of-concept studies utilizing, for example, genetically engineered models allowing tumor cell- and tissue-specific knockout or expression of conditionally targeted K_Ca_3.1 alleles in vivo.

### 2.3. Tumor Cell Apoptosis and Survival

Besides cell cycle progression, the cell cycle control machinery acts to avoid mitotic abnormalities and thereby DNA damage. The latter triggers either its repair or programed cell death ensuring that cells with damaged DNA cannot pass on their genetic information. Tumor cells have developed escape strategies allowing them to avoid cell cycle control and cell death by different mechanisms [81]. In various types of tumor cells, K_Ca_3.1 signaling appears to interfere with apoptotic cell death triggered by transmembrane death receptor and mitochondrial pathways [73,82,83]. Moreover, apoptotic cell death of poorly differentiated triple-negative breast cancer cells was promoted by TRAM-34 [84]. In different melanoma cell lines, TRAM-34 by itself did not elicit apoptosis; however, when applied together with tumor-necrosis-factor-related-apoptosis-inducing-ligand (TRAIL), it stimulated the mitochondrial release of cytochrome c, thereby triggering a cascade of caspase activation. Intriguingly, agonistic TRAIL death receptor expression was found to be upregulated by TRAM-34 suggesting that K_Ca_3.1 plays a key role in sensitizing melanoma cells to TRAIL-induced apoptosis [85]. K_Ca_3.1 channel expression in mitochondria was shown for the HCT116 human colon carcinoma cell line and in HeLa cells where K_Ca_3.1-specific siRNA induced the release of apoptosis-initiating mediators of the intrinsic pathway [78,86]. Apparently, K_Ca_3.1 is also involved in K^+^ flux across the mitochondrial membrane in tumor cells. As a classical stimulator of the intrinsic apoptotic pathway, staurosporine induced a Ca^2+^ signal in D54-MG glioma cells that triggered plasma membrane K^+^ efflux via K_Ca_3.1 resulting in caspase-3 activation and apoptotic volume decrease [83]. In contrast, caspase-3 activity after cisplatin treatment was inhibited by K_Ca_3.1 blockade and amplified under 1-EBIO in epidermoid cancer cells [87]. K_v_1.3, a voltage-gated K^+^ channel that amongst others associates with K_Ca_3.1 for immune activation [88], was already shown to induce mitochondria-dependent apoptosis in lymphocytes [89] but also in cancer cell lines as well as in vivo melanoma and pancreatic cancer models [90,91]. In our previous work, we observed increased histone 2AX (H2AX) phosphorylation on serine 139, an indicator for DNA damage, after irradiating T98G and U87MG glioma cells co-treated with TRAM-34. As K_Ca_3.1 inhibition increased radiosensitivity of glioma cells in vitro and in ectopically growing gliomas in nude mice, we concluded that this channel plays a role in DNA repair processes and thereby in cell survival after radiotherapy [72,92]. In contrast, apoptosis was decreased or even abolished with K_Ca_3.1 inhibition in thymocytes and erythrocytes [93,94]. This discrepancy suggests that K_Ca_3.1 affects the programed cell death either in a cell type-specific manner across cellular differentiation processes even though the anti-apoptotic properties of K_Ca_3.1 seem to dominate.

### 2.4. Cancer Invasion and Metastasis

One of the biggest challenges in tumor therapy is the local restriction of cancer growth, since approximately 90% of all cancer patients die of secondary tumors [48,81,95]. Migration and infiltration depend on haptotactical and chemotactical signals, Ca^2+^, cell volume and intracellular signaling cascades modulating cytoskeletal dynamics [63]. K^+^ channel activity can control any of these processes. As an example, enrichment of a specific splice variant of the big conductance Ca^2+^- and voltage-activated BK K^+^ channel has been characterized as crucial factor for the pro-migratory and pro-invasive properties in glioma [96,97,98,99,100]. Likewise, K_Ca_3.1 function has been demonstrated to be required for glioma cell migration and brain infiltration [101,102,103,104,105,106,107]. Beyond, motorizing migration by locally changing the cell volume [108], BK and K_Ca_3.1 K^+^ channels are part of the Ca^2+^ signaling complex that programs glioblastoma cell migration [92,98,109,110]. Moreover, BK and K_Ca_3.1 K^+^ channels are highly expressed in stem-like subpopulations of glioblastoma [111,112,113] where they contribute to the high radioresistance [113] and pronounced migration [111,112] of these cells. Similarly to glioblastoma, K^+^ channels including K_Ca_3.1 contribute to cell migration and metastasis of extracranial tumors [63,114,115,116]. 

In particular, K_Ca_3.1 blockade or downregulation in Skov-3 human ovarian cancer cells prevented ATP-induced cell migration possibly due to a loss of interaction between K_Ca_3.1 and the purinergic receptor P2Y_2_ [117]. Charybdotoxin impaired K_Ca_3.1-mediated locomotion of human A7 and SKMEL28 melanoma cells and it decreased [Ca^2+^]_i_ and in consequence the polymerization reaction of F-actin [118]. Interfering with K_Ca_3.1 activity by different means resulted in a reduced migration rate of MDA-MB-231 breast cancer cells [84]. However, neither MDA-MB-231 cell division, migration or invasive behaviors were affected by K_Ca_3.1 overexpression or channel activation by 1-EBIO in vitro [62]. Tumor spread in vivo requires a condition of multiple interactions between malignant cells and their environment. Consistent with this understanding, a tumor-promoting microenvironment may amplify the oncogenic properties of the K_Ca_3.1.

## 3. K_Ca_3.1 in the Tumor Microenvironment

### 3.1. Tumor Stroma

Cancer-associated fibroblasts (CAF) reportedly communicate with tumor cells and other cells in order to promote tumor growth, angiogenesis, and metastasis [119]. Upregulation of the K_Ca_3.1 by growth factors, especially bFGF and to a minor extent by transforming growth factor-β, was observed in fibroblast-like cell lines, whereas the K_Ca_3.1 status of CAFs is largely unclear so far. In 10T1/2 cells, a murine embryo fibroblast cell line, growth factor-regulated K_Ca_3.1 signaling was linked to the Ras/MEK/ERK pathway and resulted in an accelerated pro-proliferative behavior but diminished myogenic differentiation [120]. In renal fibroblasts, TRAM-34 mitigated the bFGF-induced bromodeoxyuridine (BrdU) incorporation as a marker of cell cycle progression without affecting apoptosis. In a pre-clinical model of renal fibrosis, fibrotic kidneys highly upregulated K_Ca_3.1 transcripts and protein compared to sham-operated kidneys. Furthermore, fibrotic kidneys from K_Ca_3.1 knockout (KO) mice presented with less collagen deposition and fewer α-smooth muscle actin-positive cells as well as a better preservation of functional renal tissue compared to control [22]. In the angiotensin II-stimulated heart, augmented K_Ca_3.1 mRNA and protein levels promoted accumulation of cardiac fibroblasts, an effect which was fully antagonized by TRAM-34 [121,122]. In addition to these growth- and proliferation-stimulating effects, expression and release of pro-inflammatory factors such as interleukin-6 and interleukin-8, monocyte chemotactic protein 1, and matrix metalloproteinase-3 have been linked to K_Ca_3.1 function in synovial fibroblasts that derived from rheumatoid arthritis patients [123]. It needs to be determined how tumor aggressiveness is affected by K_Ca_3.1 function in CAFs. Based on the available studies from other disease models, K_Ca_3.1 in this heterogeneous cell population may have a negative impact on tumor progression and cancer therapy. 

### 3.2. Angiogenesis

In contrast to healthy tissue, growing tumors secure their nutrient and oxygen supply by the induction of angiogenesis, meaning that normally quiescent vessels sprout continuously as part of the so-called angiogenic switch. The developing blood vessels are poorly organized, immature, and not well perfused. Angiogenesis is mostly reached by unbalancing pro-angiogenic and anti-angiogenic factors like VEGF, fibroblast growth factor (FGF) or thrombospondin-1, respectively [81,95,115,124]. The exact mechanisms are poorly understood and possibly depend on the tumor entity, although VEGF and VEGF receptor inhibitors are already applied for the treatment of advanced solid tumors [81,95,125,126]. 

K^+^ channels are thought to coordinate angiogenesis by regulation of the V_m_ and [Ca^2+^]_i_ as well as by interaction with VEGF or FGF [115]. So far, K_Ca_3.1 activity has not directly been linked to tumor angiogenesis. However, abnormal levels of endothelial cell proliferation are commonly observed in the tumor vasculature [81,95] and bone marrow-derived endothelial progenitor cells expressing K_Ca_3.1 also exhibit a clotrimazole-sensitive K^+^ current [127]. Furthermore, bFGF and VEGF upregulate K_Ca_3.1 and this was essential for proliferation of HUVEC and HMEC-1 endothelial cells and angiogenesis in vivo. Importantly, bFGF-induced endothelial cell proliferation was sensitive to clotrimazole or TRAM-34, which points to K_Ca_3.1 channels as an important downstream signaling molecule. In an in vivo matrigel plug assay, continuous administration of TRAM-34 for two weeks suppressed angiogenesis in mice [13,128]. K_Ca_3.1 channels were also implicated as important regulators of endothelial cell V_m_ in human mesenteric endothelium in situ. In the same study, mesenteric arteries from patients with colon cancer showed an increase of endothelial cells expressing K_Ca_3.1 [129]. In response to epidermal growth factor (EGF), both transcriptional and protein levels of K_Ca_3.1 increased in HUVECs, whereas TRAM-34 interfered with the EGF-induced proliferation response, counteracted migration, tube formation, matrix metalloproteinase-2 upregulation and, consequently, it suppressed EGF-mediated angiogenesis in vivo [130]. Upregulation of K_Ca_3.1 promoted platelet-derived growth factor (PDGF)-induced proliferation in vascular smooth muscle cells and, conversely, its modulation by TRAM-34 attenuated the accumulation of cell proliferation markers [131]. Another characteristic of the angiogenic switch refers to bone marrow-derived cells and primarily immune cells that contribute to the building of new vessels via vasculogenesis for example by infiltrating premalignant lesions as well as progressed tumors [81,95]. The potential role(s) of cancer-associated K_Ca_3.1 channels in these cell types will be explained in more detail within the following section. In summary, further evidence is required in order to fully understand how vascular K_Ca_3.1 activity contributes to the blood supply of a tumor in vivo. 

### 3.3. The Immune System

Immune surveillance and tumor-promoting inflammation on the one hand and immune suppression in the tumor microenvironment that may result in tumor immune evasion on the other hand are hallmarks of cancer progression. Inflammation produces factors that stimulate growth and survival, angiogenesis, and epithelial-mesenchymal transition. Additionally, radicals released from immune cells may drive mutagenesis in tumor cells. With regard to immunoediting processes, highly immunogenic tumor cells are detected and eliminated, whereas survival and growth of tumor cell clones that are hardly recognized by the immune system are promoted subsequently. In this context, tumor cells can develop different strategies to avoid immune cell recognition [95]. Both innate and acquired immunity comprise complex mechanisms and a variety of cells that act in concert for rapid and successful defense against foreign and abnormal structures. To this end, immune cells circulate through the body and chemotaxis allows specific immune cell subsets to be recruited to sites of inflammation. Cells of the acquired immune response are primed against an antigen, which is driving their maturation and expansion. 

The prominent role of the K_Ca_3.1 channel for proper development and function of the immune system has been recognized by many studies over the last two decades. Together with K_v_1.3, K_Ca_3.1 channels are of crucial importance for function of the different T and B cell subsets substantiated by the notion that K_Ca_3.1 channel expression is low in naïve and memory B cells but strongly increases upon their activation [132,133,134]. In primary CD4^+^ helper T cells expressing tagged K_Ca_3.1, antigen presentation induced recruitment of K_Ca_3.1 to the immunological synapse where it was important for B cell-stimulated [Ca^2+^]_i_ increase [135]. The effector memory T cell subtype is important for the induction of a rapid secondary immune response. Interestingly, these cells show a low K_Ca_3.1 expression profile in their active state compared to naïve or the central memory T cells [136]. Similar to naïve T cells, regulatory T cells show a rather low K_Ca_3.1 expression [137]. Accordingly, in a murine model of T cell-mediated colitis, the K_Ca_3.1 KO genotype was associated with impaired Ca^2+^ influx and cytokine production of particular Th0, Th1, and Th2 subsets but had no influence on regulatory T cells or Th17 cells [138].

Moreover, Ca^2+^ oscillations-mediated TRP melastatin-7 (TRPM7) channel activity has been linked to K_Ca_3.1 function both affecting T cell migration [139]. In addition, K_Ca_3.1 seems to be necessary for the Ca^2+^-induced apoptotic volume decrease, which is followed by the appearance of phosphatidylserine at the cell surface resulting in T cell depletion [93]. Antigen-dependent differentiation of B cells and germinal center formation require function of the tissue-specific transcriptional coactivator OCA-B and *KCNN4* is one of the target genes of OCA-B. Accordingly, OCA-B KO B cells were shown to proliferate less in response to B cell receptor ligation, an effect that involved a strong OCA-B-dependent upregulation of *KCNN4* transcription [140]. Additionally, a patient of common variable immunodeficiency carried a *KCNN4* gene hypermethylation, whereas its healthy monozygotic twin had no changes regarding *KCNN4* methylation status [141]. Another example is provided by a study on chronic lymphocytic leukemia where K_Ca_3.1 mRNA and protein expression were associated with the high proliferation rate of these cells, which could be diminished by TRAM-34 [142].

Regarding antigen-presenting cells, we and others could show that the K_Ca_3.1 contributes to the migration of DCs. Sensitization and stimulation with ovalbumin increased K_Ca_3.1 protein expression in DCs, whereas their chemotaxis in response to the lymphoid chemoattractants CCL19 and CCL21 was abolished with TRAM-34. Accordingly, [Ca^2+^]_i_ raises were observed upon stimulation of the DCs with either the K_Ca_3.1 activator 1-EBIO, CCL19, or CCL21 [143]. Similarly, K_Ca_3.1 was involved in LPS-derived [Ca^2+^]_i_ increase, cell swelling and migration of bone marrow-derived DCs in mice [16]. Besides migration, the DC maturation markers CD25 and CD83 were modified by TRAM-34, however, with no impact on the ability of DCs to activate T cells [144]. In addition to antigen presentation, macrophages patrol in the body and degrade foreign structures by phagocytosis. These cells are usually stimulated by factors released by immune cells and they secrete cytokines in order to modulate inflammatory processes. Extracellular ATP, as secreted by many cells during inflammation or infection, was described to provoke K_Ca_3.1-dependent [Ca^2+^]_i_ oscillations in macrophages. In addition, macrophage stimulation by ATP resulted in transcription of the interleukin-6 (IL-6) gene [145]. Interestingly, LoVo colon cancer cell invasion was stimulated by IL-6 and IL-8, and invasiveness of these cells was enhanced in the presence of tumor-associated macrophages. Most importantly, cancer cell invasion was decreased with depletion of K_Ca_3.1 expression levels in the tumor-associated macrophages [146].

Specialized monocytic cells such as microglia, which are located throughout the brain and spinal cord, show much higher amounts of K_Ca_3.1 mRNA as compared to neurons and astrocytes. LPS stimulation of microglia did not change K_Ca_3.1 expression, but activated their neurotoxic activity, which was sensitive to TRAM-34 [147]. Microglial migration was not promoted by LPS but by IL-4, and this again was blocked by TRAM-34 [148]. In glioma, the grade of malignancy correlates with macrophage and resident microglia infiltration into the tumor and in particular with the presence of M2 macrophages. These cells do not produce pro-inflammatory cytokines, which affects the equilibrium between immune recognition and immune suppression promoting disease progression [149,150]. Along those lines, microglia cultured in glioma-conditioned medium or microglia derived from glioma-bearing mice or human biopsies polarized into an anti-inflammatory and therefore tumor-promoting phenotype. The anti-inflammatory microglia expressed high amounts of K_Ca_3.1 mRNA, and TRAM-34 switched the anti-inflammatory phenotype back to microglia with pro-inflammatory anti-tumor capacity [151].

Natural killer (NK) cells are specialized cytotoxic cells that express both K_v_1.3 and K_Ca_3.1 channels with levels independent from their maturation status. Non-adherent NK cells predominantly express K_v_1.3, whereas increased K_Ca_3.1 levels are observed in adherent NK cells. TRAM-34 reportedly promoted NK cell proliferation of adherent and non-adherent NK cells and degranulation in adherent NK cells. In vivo, TRAM-34 enhanced the anti-tumor activity of adherent, but not that of non-adherent NK cells. Regarding chemokine receptor expression essential for chemotaxis, a depletion of CX3CR1 was apparent in non-adherent NK cells in the presence of TRAM-34, whereas related receptors such as CCR1, CCR2, CCR5, CXCR3, or CXCR4 as well as cell migration were unaltered in both adherent and non-adherent NK cells [152]. Recently, K_Ca_3.1 mRNA expression was also confirmed in neutrophils, in which the K_Ca_3.1 channel affects cell volume and chemotaxis. K_Ca_3.1 KO mice showed a less effective recruitment of neutrophils to the inflammation site after LPS delivery in the airways. However, TRAM-34 did neither influence Ca^2+^ entry nor the production of reactive oxygen species in neutrophils [153]. We and others could find evidence for functional K_Ca_3.1 expression in mast cells. Based on β-hexosaminidase as well as histamine release, their degranulation was dependent on K_Ca_3.1 channels. Accordingly, in vivo analysis of K_Ca_3.1 KO mice revealed a lower antigen-provoked decline in body temperature upon IgE challenge as a measure of anaphylactic reaction, when compared to the wildtype mouse [17,154]. Finally, human mast cell migration towards different chemoattractants, but not their proliferation rate, declined with pharmacological K_Ca_3.1 blockade [155]. 

**Figure 2:**
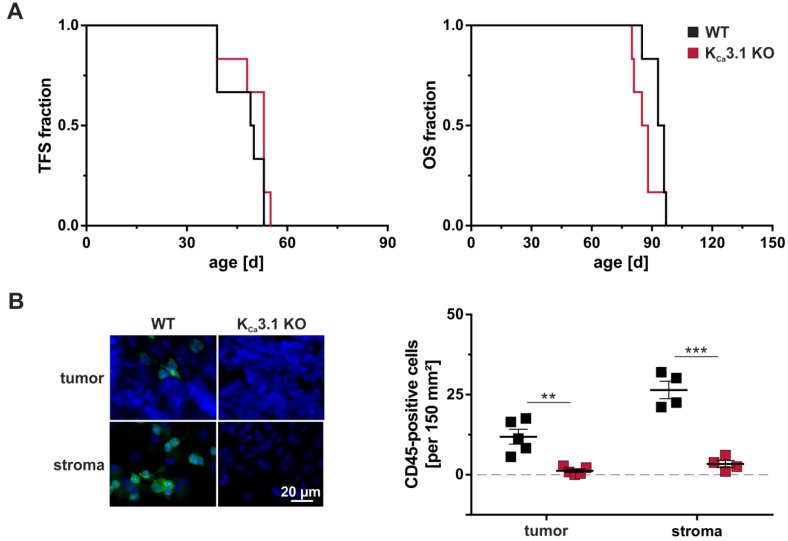
Tumorigenesis, progression, and CD45 status in MMTV-PyMT WT and K_Ca_3.1 KO mice. Tumor-free survival (TFS) and overall survival (OS) were studied in spontaneous breast cancer-prone MMTV-PyMT wildtype (WT) and K_Ca_3.1 KO mice. At a diameter of 15 mm, tumors were harvested for investigating immune cell infiltration. (**A**) As previously reported [71], tumorigenesis and tumor progression measured by TFS and OS, respectively, were not dependent on MMTV-PyMT WT or K_Ca_3.1 KO genotypes (*n* = 6 each). (**B**) Staining against the CD45 pan leukocyte marker revealed moderate immune cell infiltration in WT tumors (green), which was absent in K_Ca_3.1 KO. Immune cells were generally more abundant in the tumor-surrounding stroma of WT mice, but mostly absent in K_Ca_3.1 KO tumor samples. DAPI labelling was performed to visualize nuclei. Results are presented as means ± SEM for *n* = 4 stroma sections and *n* = 5 tumor sections of MMTV-PyMT WT (black squares) or K_Ca_3.1 KO (red squares) genotypes. Unpaired *t*-tests differentiated between groups with ** *p* < 0.01 and *** *p* < 0.001.

A comprehensive analysis of peripheral blood revealed no significant difference in blood cell counts between K_Ca_3.1 KO and wildtype mice. Cells tested included erythrocytes as well as total and differential leukocyte count including lymphocytes, eosinophils, neutrophils and monocytes [153]. Importantly, CD19^+^ B cells, CD4^+^, and CD8^+^ T cells as well as CD4^+^CD25^+^FOXP3^+^ cells representing regulatory T cells were not altered by the absence of K_Ca_3.1 [138]. In our recently investigated MMTV-PyMT breast tumor model [71] (Figure 2A), we identified breast tumor-infiltrating leukocytes by using the CD45 pan leukocyte marker. Consistently, a much higher number of CD45^+^ cells was present in the tumor-surrounding stroma as compared to the tumor itself (Figure 2B). In tumor sections derived from MMTV-PyMT K_Ca_3.1 KO mice, however, CD45^+^ cells were not detected in the tumor and very rare in the stroma. Together, these data support the notion that inadequate levels of K_Ca_3.1 activity, although not affecting total and differential leukocyte count in vivo, show influence on immune cell maturation and thereby perturb a proper immune cell infiltration of the tumor. 

### 3.4. Anti-Cancer Therapy with K_Ca_3.1 Modulators

As shown with numerous examples in the previous sections, K_Ca_3.1 inhibition is a powerful approach to interact with malignant cell cycle progression and thus tumor growth, cell migration, and other tumor-promoting features. The pharmacology of the different K_Ca_3.1 inhibitors including the new benzothiazone NS6180 [156] or the most commonly used inhibitors—clotrimazole, TRAM-34, and senicapoc—which are described in more detail in the following sections, is well-known.

Clotrimazole (1-[(2-Chlorphenyl)diphenylmethyl]-1H-imidazol) is a small molecule, which was primarily designed as an anti-mycotic drug. However, further investigations indicated an inhibition of various cytochrome P450 enzymes, in particular CYP3A4 [157], and blockade of the K_Ca_3.1 channel with an IC_50_ of 70 nM [156,158]. As already mentioned in the previous sections, clotrimazole reduces cell proliferation in a dose-dependent manner in e.g., human melanoma and glioblastoma cell lines [159,160]. Although clotrimazole has inhibitory effects on cancer cells, other inhibitors not interfering with the cytochrome P450 system and more selective for K_Ca_3.1 should be preferred in experimental and pre-/clinical research [59,161]. 

One of these agents with improved properties is TRAM-34 (1-[(2-chlorophenyl) diphenylmethyl]-1H-pyrazole) [84], which is a modified triarylmethane pyrazole analogue of the clotrimazole molecule [59,80,134]. TRAM-34 is currently not in clinical use, but indicates a solid K_Ca_3.1 affinity in animals like rats and mice (IC_50_ 20-25 nM) [80,156,162]. Depending on its concentration, TRAM-34 can almost completely inhibit tumor proliferation of pancreatic ductal adenocarcinoma cells and many other tumor cell types as outlined in the previous chapters of this review [35,163,164]. Senicapoc or ICA-17043 (2,2-bis(4-fluorophenyl)-2-phenylacetamide) is similar to TRAM-34 in its chemical structure, but has a higher affinity for K_Ca_3.1 (IC_50_ 11 nM) [165,166]. An advantage is the oral bioavailability of senicapoc, whose half-life of 12.8 days is also much higher than that of TRAM-34 (2 h) [165,167,168]. So far, senicapoc has been mostly investigated as a possible drug in sickle cell anemia treatment, i.e., phase I and II clinical studies on safety and efficacy have been completed [169]. Senicapoc went into randomized phase III clinical trials where it showed beneficial effects by decreasing certain disease markers, e.g., lactate dehydrogenase and bilirubin. Despite these promising effects, the study was terminated ahead of schedule due to a lack of efficacy in the patient cohort [169,170]. Besides, senicapoc has been proven as effective K_Ca_3.1 inhibitor in experimental cancer research. After a six-day therapy in vivo, intrahepatic cholangiocarcinoma tumor volume and weight of the mice were significantly reduced [164]. Together with its safety profile, further research with senicapoc in cancer is indicated.

Besides K_Ca_3.1 inhibition, its activation especially with regard to immune cell functions in general and in the immune cell’s control of malignant diseases also needs further exploration. The classic K_Ca_3.1 channel opener is 1-EBIO (1-ethyl-2-benzimidazolinone) (EC_50_ 30 µM), which was first described in 1996 [171,172]. It shows a proper effect on some Ca^2+^-activated K^+^ channels and could for example rescue ionomycin-induced cell death in head and neck squamous cell carcinoma cells shown by Yin et al. [173]. More potent analogues are DC-EBIO (5,6-dichloro-1-ethyl-1,3-dihydro-2H-benzimidazol-2-one) (EC_50_ 1 µM) [174], NS-309 (3-Oxime-6,7-dichloro-1H-indole-2,3-dione) (EC_50_ 20 nM) [172] and SKA-31 (naphtho[1,2-d]thiazol-2-ylamine) (EC_50_ 250 nM) [175]. Except for SKA-31, all these compounds are not completely K_Ca_3.1-selective [176] and need a minimum of Ca^2+^ to be effective [171,175,177]. Interestingly, clotrimazole and TRAM-34 could abolish 1-EBIO-induced cell proliferation in hepatocellular and in prostate cancer cells [58,178].

## 4. Conclusions

It has only recently been recognized that K_Ca_3.1 contributes to the malignant cell behaviors seen in cancer. Based on the available data, “oncochannels” such as K_Ca_3.1 as well as dysregulated signaling pathways that depend on these channels may be promising candidates in the therapy of various solid tumors including, among others, glioblastoma, endometrial, prostate, breast, hepatocellular, and cervical carcinoma. Such considerations seem justified, as an effective inhibition of K_Ca_3.1 by genetic and pharmacological means markedly reduces the proliferation of tumor cells and it may also alter the susceptibility of the tumor towards established cancer therapies. In this respect, senicapoc, which proved safe in clinical trials on sickle cell anemia, represents a repurposable candidate drug for future investigations into the anti-tumor action of K_Ca_3.1 inhibition. So far, targeting of K_Ca_3.1 by senicapoc is suggested in combination with existing chemoradiotherapy regimes to tackle the therapy-resistant cancer (stem) cells [102,109,113,179].

**Figure 3:**
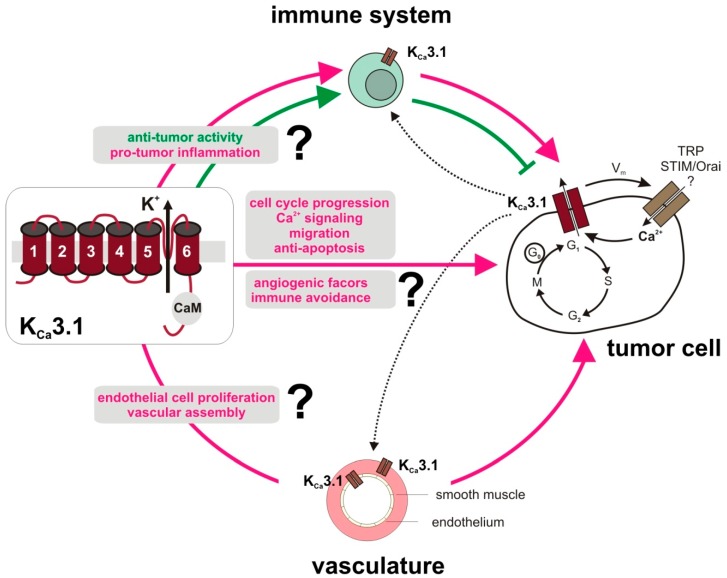
Role of the K_Ca_3.1 channel in tumor-associated cells. Tumors from different entities and various microenvironmental cell types, i.e., immune cells, vasculature, fibroblasts (not shown) express functional K_Ca_3.1 channels. Physiological roles and tumor behaviors of K_Ca_3.1 are cell type-dependent, but involve proliferation, migration and cancer progression. K_Ca_3.1 channel expression seems to be a crucial determinant of cancer risk and, in established cancers, K_Ca_3.1 upregulation at the end of G_1_ phase of the cell cycle was seen in various tumor cell types [36]. By its interaction with [Ca^2+^]_i_ via its constitutively bound calmodulin (CaM), with other ion channels such as TRP or STIM/Orai, with changes in the membrane potential (V_m_), and with apoptotic pathways, K_Ca_3.1 may further contribute to aberrant tumor cell signaling. Beyond that, tumor-promoting K_Ca_3.1 activity in stromal cells has been described. Several studies find evidence for K_Ca_3.1 expression in endothelial and in activated smooth muscle cells of the vasculature pointing to its role in tumor angiogenesis and/or vasculogenesis. Moreover, growth factor signaling was linked to K_Ca_3.1 in fibroblasts to promote epithelial-mesenchymal transition in breast cancer (not depicted) [84]. Proper activation and function of various immune cell subsets requires K_Ca_3.1. Therefore, perturbed K_Ca_3.1 signaling may prevent cancer progression and disturb e.g., the immune cell´s pro-angiogenic program, but also its activity to recognize and eliminate tumor cells. Apparently, the impact of a tumor and stromal versus immune cell K_Ca_3.1 inhibition on tumor progression and therapy success and thus also interaction between the different cell types, as indicated by dotted lanes, requires further investigations.

Besides adverse effects of K_Ca_3.1 action stemming from the tumor cell itself, K_Ca_3.1 might also be important for the supply of the tumor with pro-tumorigenic factors from cells interacting with the tumor. To adequately and accurately meet the specific challenges of cancer, it will be necessary to better characterize the tumor environment with respect to K_Ca_3.1 channel functions in stromal cell types, tumor microvasculature, and in the immune system. A dysregulation of K_Ca_3.1 in the latter may impair both detection and destruction of aberrant cells, which is in support of the establishment of a tumor in its niche rather than its elimination. Paradoxically, tumor cells that acquire the ability to escape immune recognition further progress by responding to pro-inflammatory factors secreted from invading immune cells. Therefore, it is also tempting to speculate that K_Ca_3.1 inhibition might delay the progression of such immune-evaded tumors (Figure 3). 

Finally, we should make every effort to intensify the genomic/epigenomic and transcriptomic profiling of putative “oncochannels” within population- and patient-based screenings because this will allow estimations if and how these channels affect cancer development and progression, as well as sensitivity to drug treatment. To this end, such comprehensive data sets should provide important information for the prediction of patient outcome in order to facilitate personalized drug treatments.
